# Low concentrations of clarithromycin upregulate cellular antioxidant enzymes and phosphorylation of extracellular signal-regulated kinase in human small airway epithelial cells

**DOI:** 10.1186/s40780-018-0120-4

**Published:** 2018-09-03

**Authors:** Kuninori Iwayama, Junpei Kimura, Aya Mishima, Ayuko Kusakabe, Ko-ichi Ohtaki, Yoshiko Tampo, Nobumasa Hayase

**Affiliations:** 1 Department of Pharmacology and Therapeutics, Hokkaido Pharmaceutical University School of Pharmacy, 7-15-4-1 Maeda, Teine, Sapporo, Hokkaido 006-8590 Japan; 2 Department of Hospital Pharmacy and Pharmacology, Asahikawa Medical University Hospital, Asahikawa, 078-8510 Japan; 3 Department of Pharmacy, Nakamura Memorial Hospital, Sapporo, 060-8570 Japan; 4 Department of Pharmacy, Kushiro Kojinkai Memorial Hospital, Kushiro, 085-0062 Japan; 5 Department of Pharmacy, Shin-Sapporo Towakai Hospital, Sapporo, 004-0041 Japan; 6 Department of Public and Health, Hokkaido Pharmaceutical University School of Pharmacy, Sapporo, 006-8590 Japan

**Keywords:** Clarithromycin, Anti-inflammatory effect, Low-dose, Long-term treatment, Human small airway epithelial cells, Oxidant/antioxidant balance, Cellular antioxidant enzyme, Extracellular signal regulatory kinase

## Abstract

**Background:**

It is well known that low-dose, long-term macrolide therapy is effective against chronic inflammatory airway diseases. Oxidative stress is considered to be a key pathogenesis factor in those diseases. However, the mechanism of action of low-dose, long-term macrolide therapy remains unclear. We have reported that clarithromycin (CAM), which is a representative macrolide antibiotic, could inhibit hydrogen peroxide (H_2_O_2_)-induced reduction of the glutathione (GSH)/glutathione disulfide (GSSG) ratio in human small airway epithelial cells (SAECs), via the maintenance of GSH levels through an effect on γ-glutamylcysteine synthetase (γ-GCS) expression. In this study, we examined the influence of CAM against H_2_O_2_-induced activities of cellular antioxidant enzymes and phosphorylated extracellular signal regulatory kinase (p-ERK) using SAECs, the main cells involved in chronic airway inflammatory diseases.

**Methods:**

SAECs were pretreated with CAM (1, 5, and 10 μM) for 72 h, and subsequently exposed to H_2_O_2_ (100 μM) for 0.5–2 h. Levels of GSH and GSSG, and activities of glutathione peroxidase (GPx)-1, glutathione reductase (GR), superoxide dismutase (SOD), catalase (CAT), heme oxygenase (HO)-1 and p-ERK were assayed. mRNA expressions of GPx-1 and HO-1 were measured using the real-time reverse transcription polymerase chain reaction (RT-PCR). Tukey’s multiple comparison test was used for analysis of statistical significance.

**Results:**

Pretreatment with low-dose (1 and 5 μM) CAM for 72 h inhibited H_2_O_2_-induced reductions of GPx-1, GR, SOD, CAT and HO-1 activities, and mRNA expressions of GPx-1 and HO-1, and improved the GSH/GSSG ratio. However, these alterations were not observed after pretreatment with high-dose (10 μM) CAM, which suppressed phosphorylation of cell proliferation-associated ERK to cause a significant (*p* < 0.01) decrease in cell viability.

**Conclusions:**

CAM is efficacious against deterioration of cellular antioxidant enzyme activity caused by oxidative stress under low-dose, long-term treatment conditions. On the other hand, pretreatment with high-dose CAM suppressed phosphorylation of cell proliferation-associated ERK and decreased cell viability. The present study may provide additional evidence as to why low-dose, long-term administration of macrolides is effective for treating chronic inflammatory airway diseases.

**Electronic supplementary material:**

The online version of this article (10.1186/s40780-018-0120-4) contains supplementary material, which is available to authorized users.

## Background

Macrolides such as clarithromycin (CAM) have been reported to be effective for the treatment of chronic inflammatory airway diseases at low doses and with long-term administration [1–5]. The effectiveness of macrolides in treating inflammatory airway diseases has been thought to be due to their immunomodulatory effects rather than their direct antimicrobial activity. For instance, both CAM and erythromycin (EM) inhibit the production of inflammatory cytokines, such as interleukin (IL)-6 and IL-8, inhibit the release of soluble intracellular adhesion molecule-1 from airway epithelial cells, and decrease airway neutrophil accumulation [6, 7]. However, there is limited data concerning the potential benefits of low-dose, long-term application of CAM in a variety of chronic inflammatory airway diseases.

**Scheme 1:**
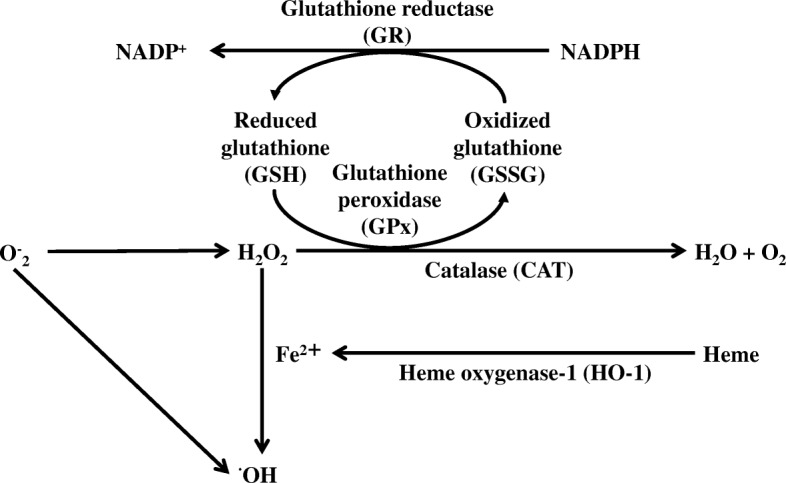
Cellular antioxidant enzymes involved in the ROS elimination system

Recently, we demonstrated that long-term pretreatment with low-dose CAM is effective for suppressing the expression of transcription factors involved in inflammatory cytokine production in response to hydrogen peroxide (H_2_O_2_)-induced cytotoxicity in human small airway epithelial cells (SAECs) [8]. For example, pre-treatment with 1 or 5 μM CAM, but not with 10 μM CAM, for 72 h prior to H_2_O_2_ treatment significantly decreased H_2_O_2_-induced IL-8 protein release (see Additional file 1). H_2_O_2_ is one of the reactive oxygen species (ROS), and was employed in this study to alter the oxidant/antioxidant balance in living cells. In chronic inflammatory respiratory diseases such as chronic obstructive pulmonary disease (COPD) [9], bronchial asthma [10], bronchiectasis [11] and cystic fibrosis [12], the H_2_O_2_ concentration in the exhaled breath has been reported to be several times higher than healthy subjects. Therefore, in these pathological conditions, it may be considered that the oxidant/antioxidant balance in bronchial epithelial cells is seriously impaired. Indeed, our previous study demonstrated that the intracellular glutathione (GSH)/glutathione disulfide (GSSG) ratio is strongly reduced when SAECs are treated with H_2_O_2_ (100 μM) [8]. However, long-term (72 h) pretreatment with low-dose (1 or 5 μM) CAM significantly improved this ratio to maintain cell viability (see Additional file 2). In contrast, this alteration was not observed after pretreatment with high-dose (10 μM) or short-term (24 and 48 h) CAM. Thus, the effects of CAM on the oxidant/antioxidant balance in cells depend on the concentration and pretreatment time. On the other hand, there is a defensive pathway called the ROS elimination system that is known to suppress the concentration of ROS in cells [13] (Scheme 1). Namely, superoxide anion radicals produced in vivo by inflammatory substances is directly converted to H_2_O_2_ by superoxide dismutase (SOD). H_2_O_2_ is decomposed into water and oxygen by catalase (CAT) and glutathione peroxidase (GPx)-1, which oxidizes GSH to GSSG. Alternatively, GSSG is reduced to GSH by glutathione reductase (GR) and NADPH. Furthermore, a part of H_2_O_2_ undergoes the Fenton reaction with ferrous iron, and is converted to hydroxyl radical, which has the highest oxidizing activity. Ferrous iron is produced by the degradation of heme, and this reaction is catalyzed by heme oxidase (HO)-1. Moreover, HO-1 potentially generates a significant amount of H_2_O_2_, which is a source of hydroxyl radical. CAM may enhance the system for ROS elimination in cells. However, there are no reports describing the direct effects of CAM on activities concerning ROS elimination enzymes.

In addition to the above data, it has been observed that H_2_O_2_ activates the mitogen activated protein kinase (MAPK) pathway [14–16]. This pathway consists mainly of three types of cascade, extracellular signal regulatory kinase (ERK), c-jun N-terminal kinase (JNK) and p38 cascades. When the MAPK (ERK, JNK and p38) pathway is stimulated by oxidative stress, each of the cascades is activated by phosphorylation to exert a different physiological action [17]. Among these cascades, ERK phosphorylation mediates cell proliferation in bronchial epithelial cells [18, 19]. Therefore, it may be considered that inhibition of ERK phosphorylation causes cell death in bronchial epithelial cells. The cytoprotective action of CAM may be associated with the increase in phosphorylation against H_2_O_2_-induced cell dysfunction. However, there are no reports describing the direct effects of CAM on ERK phosphorylation induced by H_2_O_2_.

In this study, we examined the effect of CAM on H_2_O_2_-induced expression of GPx-1, GR, SOD, HO-1 and CAT in SAECs under the same conditions previously demonstrated to show the cytoprotective effect of CAM [8]. In addition, we investigated the effect of CAM on H_2_O_2_-induced ERK phosphorylation in SAECs.

## Methods

### Materials

CAM, H_2_O_2_ (30%), dimethylsulfoxide (DMSO), NaN_3_, the WST-8 (2-(2-methoxy-4-nitrophenyl)-3-(4-nitrophenyl)-5-(2,4-disulfophenyl)-2H-tetrazolium) assay system, and 5,5′-dithiobis(2-nitrobenzoic acid) (DTNB) were purchased from Wako Pure Chemical Industries, Ltd. (Osaka, Japan). Mouse anti-phosphorylated ERK and anti-total ERK monoclonal antibodies, and U0126 were from Cell Signaling Technology (Tokyo, Japan). GSH, GSSG, EDTA and mouse anti-β-actin monoclonal antibody were from Sigma-Aldrich Chemical Co. (St. Louis, MO, USA). GR (from yeast) and NADPH were from Oriental Yeast Co., Ltd. (Tokyo, Japan). Dulbecco’s phosphate-buffered saline (DPBS) and phosphate-buffered saline (PBS) at pH 7.4 were from Gibco BRL (Grand Island, NY, USA). Triton-X was from IBI Scientific (Kapp Court Peosta, IA, USA). All other chemicals used were of reagent grade.

### Cell culture and treatments

Cell culture and treatments were according to methods described previously [8]. Briefly, SAECs, cells of a normal human small airway epithelial cell line, and SAEC culture media were purchased from Lonza (Walkersville, MD, USA). Cells were seeded in 75-cm^2^ filter vent flasks (Corning, NY, USA) and grown to 80% confluence (3 × 10^6^ cells/well) for each experimental condition, at 37 °C in a humidified atmosphere of 5% CO_2_ and 95% air. The culture medium was changed on day 1 and subsequently every 2 days. Cells were passaged by trypsinization, and cultures between passages 3 and 9 were used for all experiments. Cells were cultured in the presence or absence of CAM (1, 5 or 10 μM) for 72 h and then stimulated with H_2_O_2_ (100 μM) for an additional 0.5–2 h. Neither 1, 5 μM (low) or 10 μM (high) CAM nor 100 μM H_2_O_2_ affected SAEC proliferation or viability (see Additional files 3 and 4). Based on our previous study [8], the long-term treatment with CAM was set at 72 h. Pretreatments with CAM were carried out with a different set of cells for each concentration. In addition, cell stimulation with H_2_O_2_ was performed as follows in order to determine the point at which the effect of H_2_O_2_ treatment was the strongest. For stimulation of the cells with H_2_O_2_, the medium was changed to small airway basal medium (SABM) containing no supplements, since bovine pituitary extract and serum may include antioxidants, chelators of transition metal ions, and high-density lipoproteins [20]. CAM was dissolved in DMSO at a final concentration of 10 mM and then diluted with distilled water or culture medium to the desired concentrations. The final concentration of DMSO was less than 0.1%. CAM solution used for experiments was prepared immediately before use.

### GPx-1 activity

GPx-1 activity was measured using NADPH consumption as an index [21]. SAECs (5 × 10^5^ cells/well) on 12-well plates were pretreated with CAM (1, 5 or 10 μM) for 72 h and then stimulated with H_2_O_2_ (100 μM) for 1.5 h. Cells were washed with DPBS and then solubilized with PBS (220 μL) containing 0.1% Triton-X and incubated at − 20 °C for 10 min. After incubation, the cell lysate was centrifuged at 10,000×*g* for 10 min at 4 °C. GPx-1 activity in the cell lysate was measured spectrophotometrically using a method based on the decrease in absorbance at 340 nm due to the oxidation of NADPH in the presence of GSH and GR. This assay system consisted of 50 mM PBS (pH 7.6, 150 μL) containing 1 mM NaN_3_, 1 mM EDTA, 1 mM GSH, 0.2 mM NADPH, 1 U/mL GR, sample (50 μL), to which H_2_O_2_ (250 μM) was added to start the reaction. GPx-1 activities were calculated using the molar extinction coefficient value at 340 nm of 6.22 mM^− 1^ cm^− 1^, and are expressed as a ratio (%) to changes in H_2_O_2_ untreated cells.

### Real-time RT-PCR for GPx-1 and HO-1 mRNAs

The mRNA expressions of GPx-1 and HO-1 were measured by quantitative RT-PCR analysis. Briefly, SAECs (10^6^ cells/well) in 6-well plates were pretreated with CAM (1, 5 or 10 μM) for 72 h and then stimulated with H_2_O_2_ (100 μM) for 1 h. Total RNA was obtained using a PureLink RNA Mini Kit (Life Technologies Corp., Carlsbad, CA, USA) following the manufacturer’s instructions and quantified by absorbance measurement at 260 nm. RNA (2 μg) was reverse transcribed into complementary deoxyribonucleic acid (cDNA) using a SuperScript VILO cDNA Synthesis Kit following the manufacturer’s instructions (Invitrogen, Carlsbad, CA, USA). TaqMan polymerase chain reaction (PCR) primers and probes for GPx-1 or HO-1 and for glyceraldehyde-3-phosphate dehydrogenase (GAPDH) as the internal standard gene were purchased from Applied Biosystems (Foster City, CA, USA). TaqMan PCR was performed with 1 μL of sample cDNA in a 20-μL reaction mixture containing TaqMan gene master mix and TaqMan gene expression assays for GPx-1 and HO-1. Amplification was performed using the 7500 Real Time Reverse Transcription-PCR System (Applied Biosystems). The PCR thermal protocol consisted of 50 °C for 2 min and 95 °C for 10 min, followed by 40-cycle amplification at 95 °C for 15 s and 60 °C for 1 min. Relative quantification of gene expression was performed using the comparative threshold method. Changes in mRNA expression were calculated after normalizing to GAPDH, and are expressed as a ratio to changes in H_2_O_2_ untreated cells.

### GR activity

GR activity was also measured using NADPH consumption as an index [21]. Cell pretreatment with CAM, H_2_O_2_ treatment, and sample preparation were carried out in the same manner as for measurement of GPx-1 activity. GR activity in the cell lysate was measured spectrophotometrically using a method based on the decrease in absorbance at 340 nm due to the oxidation of NADPH in the presence of GSSG. This assay system consisted of 50 mM PBS (pH 7.6, 250 μL) containing 2 mM EDTA, 1 mM GSSG, 0.1 mM NADPH and sample (100 μL). GR activities were calculated using the molar extinction coefficient value at 340 nm of 6.22 mM^− 1^ cm^− 1^, and are expressed as a ratio (%) to changes in H_2_O_2_ untreated cells.

### CAT activity

CAT activity was measured using a catalase assay kit (Cayman Chemical Co., Ann Arbor, MI, USA) according to the manufacturer’s instructions. Cell pretreatment with CAM, H_2_O_2_ treatment, and sample preparation were carried out in the same manner as for measurement of GPx-1 activity. This assay system consisted of 100 mM PBS (pH 7.0, 100 μL), methanol (30 μL) and sample (20 μL). The reaction was started by adding 35 μM H_2_O_2_ and the reaction mixture was incubated for 20 min at room temperature. After incubation, 10 M potassium hydroxide and chromogen were added to the mixture. After further incubation for 10 min, potassium periodate was added and incubated for 5 min at room temperature before reading the absorbance at 540 nm using a plate reader (Bio-Rad, Hercules, CA, USA). CAT activities were calculated using the equation obtained from the linear regression of the standard curve. Data are expressed as a ratio (%) to changes in H_2_O_2_ untreated cells.

### SOD activity

The SOD assay was performed using an SOD assay kit-WST (Dojindo Laboratories, Kumamoto, Japan) according to the manufacturer’s instructions. Cell pretreatment with CAM, H_2_O_2_ treatment, and sample preparation were carried out in the same manner as for measurement of GPx-1 activity. This assay system, based on the NBT method utilizing the tetrazolium salt reduction reaction, consisted of a sample (20 μL), the provided WST working solution (200 μL) and an enzyme working solution (20 μL). This mixture was incubated for 20 min at room temperature and absorbance was then measured at 450 nm. The SOD activities were calculated using the equation obtained from the regression analysis of the standard curve. Data are expressed as a ratio (%) to changes in H_2_O_2_ untreated cells.

### Western blot analysis for HO-1 protein and ERK phosphorylation

Phosphorylated ERK (p-ERK), total ERK (t-ERK) and HO-1 protein levels were analyzed by Western blot analysis. SAECs (10^6^ cells/well) on 6-cm dishes were pretreated with CAM (1, 5 or 10 μM) for 72 h and then treated with H_2_O_2_ for 0.5 h. Cells were washed with DPBS, and collected using fresh DPBS and a cell scraper. After centrifugation at 2000×*g* for 10 min, 50 μL of radioimmunoprecipitation assay (RIPA) buffer (Pierce, Rockford, IL, USA) containing 1 M vanadate and protease inhibitors was added and then cell pellets were sonicated three times for 10 s each. The lysates were centrifuged at 12000×*g* for 10 min, and a 10-μg aliquot of total protein in supernatant was resuspended in the same amount of sample buffer (Laemmli sample buffer containing 0.5 mM 2-mercaptoethanol) and boiled for 5 min. After refrigeration, the sample was separated using 12% sodium dodecyl sulfate polyacrylamide gel electrophoresis (SDS-PAGE). Next, the gel was electrotransferred onto a nitrocellulose membrane (Bio-Rad). Membranes were blocked in 5% nonfat dry milk in Tris-buffered solution containing 0.1% Tween 20 (TBST) at room temperature for 2 h. The membrane was incubated with a 1:2000 dilution of primary antibody for p-ERK, t-ERK or β-actin with a 1:1000 dilution of horseradish peroxidase-conjugated secondary antibody. Signals were detected as the intensity of chemiluminescence using an ECL Plus Western Blotting Detection Kit (GE Healthcare, Buckinghamshire, UK). p-ERK or HO-1 levels were normalized to constitutive expression of total protein or β-actin, respectively, and are expressed as p-ERK/t-ERK or HO-1/β-actin calculated as the scan unit ratio (%) ± standard deviation (SD) of three experiments using imageJ software (NIH, Bethesda, MD, USA).

### Cell viability

Cell viability was assessed using the cell counting kit-8 assay, WST-8. SAECs (3 × 10^4^ cells/well) on 96-well plates were pretreated with U0126 (10 μM) for 0.5 h and then stimulated with H_2_O_2_ (100 μM) for 3 h. After the treatment, the medium of SAECs was changed to basal medium (SABM) containing 10% WST-8 solution and the cells were incubated at 37 °C for 2 h. Viable cells convert the WST-8 solution to an orange-colored formazan product with an absorbance at 450 nm. The optical density of the culture medium was measured at 450 nm with a spectrophotometric microliter plate reader (Bio-Rad). The cell proliferation and viability are expressed as the ratio (%) of surviving cells to H_2_O_2_ untreated cells. The morphology of SAECs was evaluated visually throughout the course of the experiments.

### Other procedures

Intracellular GSH and GSSG levels were measured by spectrophotometric methods as described previously [8]. Protein concentrations were determined using the Bradford method with bovine serum albumin as the standard.

### Statistical analysis

All data are expressed as means ± SD. Statistical analysis was performed using one-way analysis of variance (ANOVA), and differences, which were estimated by Tukey’s multiple comparison test after the Goodness of fit test and Bartlett’s test, were considered to be statistically significant at *p* < 0.05.

## Results

### Effects of CAM pretreatment on the H_2_O_2_-induced GSH/GSSG ratio in SAECs

**Fig. 1:**
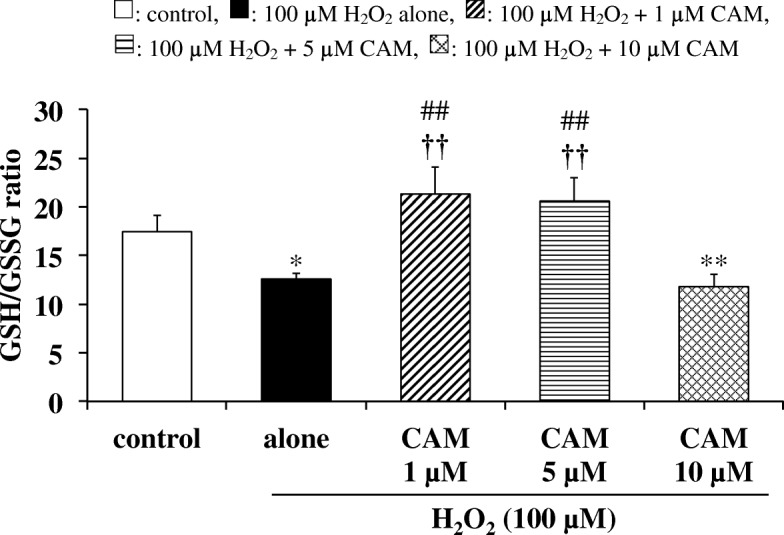
Effects of CAM pretreatment on the GSH/GSSG ratio in H_2_O_2_-stimulated SAECs. GSH and GSSG were determined using the DTNB recycling method. Samples were obtained from supernatants of control cells, of cells stimulated with 100 μM H_2_O_2_ alone, or of cells pretreated with 1, 5 or 10 μM CAM for 72 h before stimulation with 100 μM H_2_O_2_ for 2 h. Data are presented as means ± SD of three to four independent experiments. ^**^p < 0.01 vs. control cells, ^##^*p* < 0.01 vs. cells stimulated with H_2_O_2_ alone, ^††^*p* < 0.01 vs. cells pretreated with 10 μM CAM

SAECs were pretreated with CAM at 1, 5 or 10 μM for 72 h. CAM at those concentrations had no effect on cell viability (see Additional file 3). Although H_2_O_2_ treatment at 100 μM did not affect the viability of SAECs (see Additional file 4), incubation with H_2_O_2_ (100 μM) for 2 h significantly decreased the GSH/GSSG ratio compared to untreated cells (Fig. 1, *p* < 0.01). Pretreatment with a low concentration (1 or 5 μM), but not a high concentration (10 μM), of CAM for 72 h significantly increased this ratio in H_2_O_2_-treated cells (*p* < 0.01 vs. H_2_O_2_ treatment alone). There was a significant difference in the H_2_O_2_-induced GSH/GSSG ratio between the low- and high-concentration CAM groups (*p* < 0.01).

### Effects of CAM pretreatment on GPx-1, GR activities and GPx-1 mRNA expression in H_2_O_2_-treated cells

**Fig. 2:**
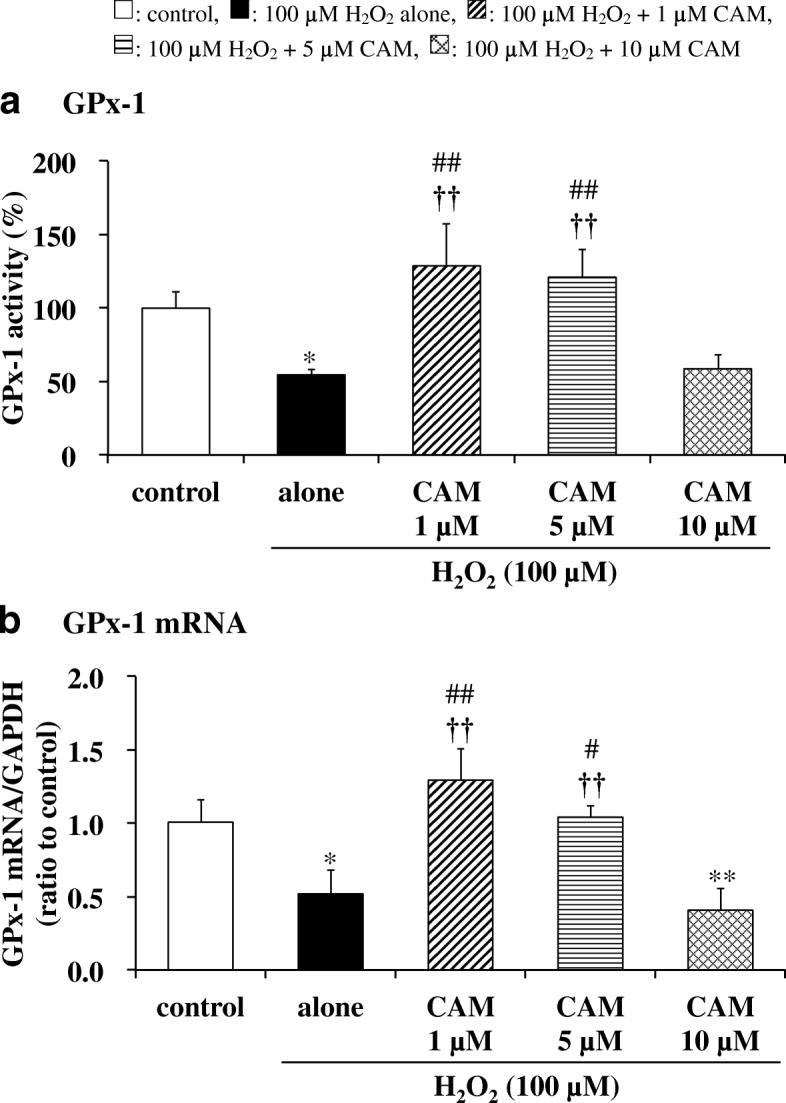
Effects of CAM pretreatment on GPx-1 activity (**a**) and mRNA expression (**b**) in H_2_O_2_-stimulated SAECs. In panel **a**, GPx-1 activities were measured using the NADPH consumption method. In panel **b**, GPx-1 mRNA expression was measured using real-time RT-PCR. Samples were obtained from supernatants (**a**) or cDNA (**b**) of control cells, of cells stimulated with 100 μM H_2_O_2_ alone, or of cells pretreated with 1, 5 or 10 μM CAM for 72 h before stimulation with 100 μM H_2_O_2_ for 1.5 or 1 h, respectively. Data are presented as means ± SD of three independent experiments. ^*^p < 0.05, ^**^p < 0.01 vs. control cells, ^#^*p* < 0.05, ^##^*p* < 0.01 vs. cells stimulated with H_2_O_2_ alone, ^††^*p* < 0.01 vs. cells pretreated with 10 μM CAM

**Fig. 3:**
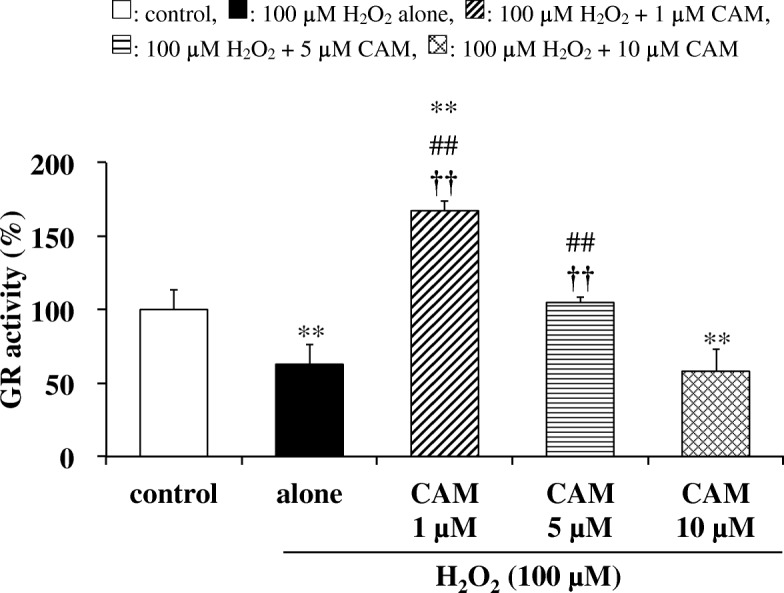
Effects of CAM pretreatment on GR activity in H_2_O_2_-stimulated SAECs. GR activities were measured using the NADPH consumption method. Samples were obtained from supernatants of control cells, of cells stimulated with 100 μM H_2_O_2_ alone, or of cells pretreated with 1, 5 or 10 μM CAM for 72 h before stimulation with 100 μM H_2_O_2_ for 1.5 h. Data are presented as means ± SD of three to five independent experiments. ^**^p < 0.01 vs. control cells, ^##^*p* < 0.01 vs. cells stimulated with H_2_O_2_ alone, ^††^*p* < 0.01 vs. cells pretreated with 10 μM CAM

GPx-1 and GR activities were significantly decreased by H_2_O_2_ treatment (100 μM) for 1.5 h compared with untreated cells (Figs. 2a and 3; *p* < 0.05 and *p* < 0.01, respectively). In contrast, pretreatment with a low concentration of CAM (1 or 5 μM) for 72 h significantly increased these activities compared with H_2_O_2_ treatment alone (*p* < 0.01). However, pretreatment with a high concentration of CAM (10 μM) for 72 h had no influence on both activities decreased by H_2_O_2_ treatment. Similar to these changes in GPx-1 and GR activities, GPx-1 mRNA expression was also significantly decreased by exposure to H_2_O_2_ for 1 h (Fig. 2b; *p* < 0.05). In contrast, a low concentration of CAM (1 or 5 μM) increased this expression compared with H_2_O_2_ treatment alone (*p* < 0.01 or *p* < 0.05, respectively). However, a high concentration of CAM (10 μM) had no effect on the expression. Similar to the effect of CAM on the H_2_O_2_-induced GSH/GSSG ratio, there were also significant differences in the H_2_O_2_-induced GPx-1, GR activities and GPx-1 mRNA expression between the low- and high-concentration CAM groups (*p* < 0.01).

### Effects of CAM pretreatment on CAT and SOD activities in H_2_O_2_-treated cells

**Fig. 4:**
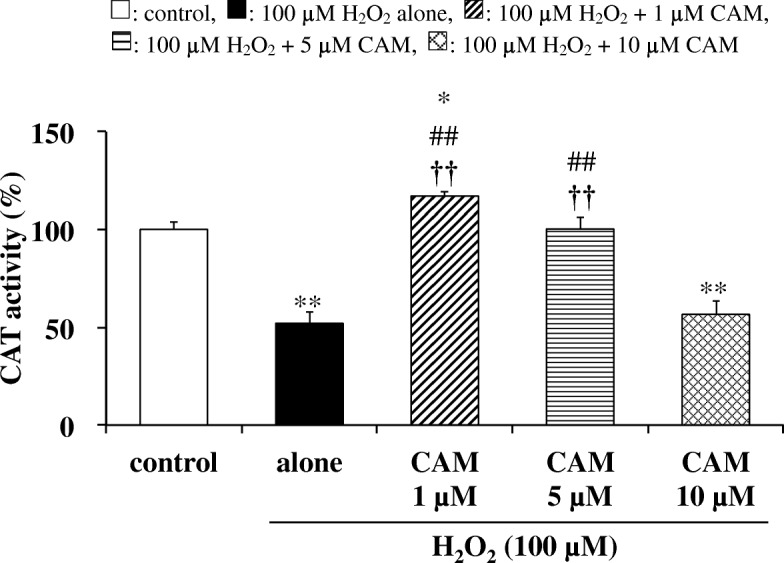
Effects of CAM pretreatment on CAT activity in H_2_O_2_-stimulated SAECs. CAT activities were measured using a catalase assay kit as described in the Methods. Samples were obtained from supernatants of control cells, of cells stimulated with 100 μM H_2_O_2_ alone, or of cells pretreated with 1, 5 or 10 μM CAM for 72 h before stimulation with 100 μM H_2_O_2_ for 1.5 h. Data are presented as means ± SD of three to six independent experiments. ^*^p < 0.05, ^**^p < 0.01 vs. control cells, ^##^*p* < 0.01 vs. cells stimulated with H_2_O_2_ alone, ^††^*p* < 0.01 vs. cells pretreated with 10 μM CAM

**Fig. 5:**
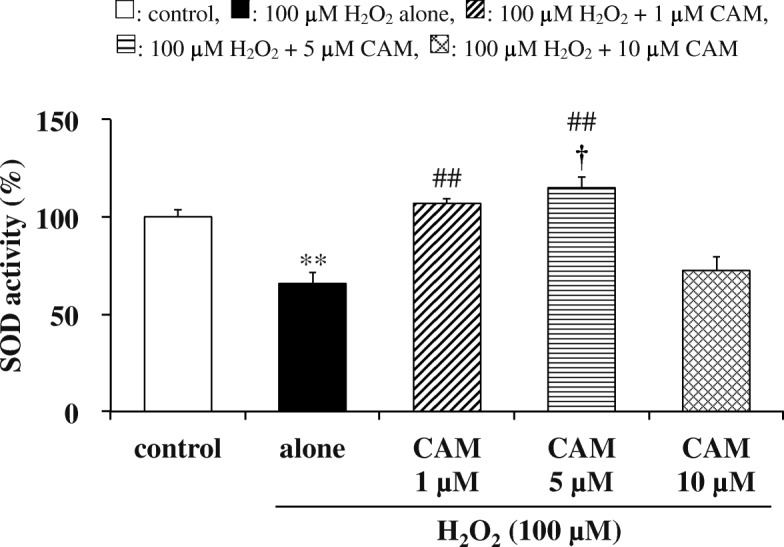
Effects of CAM pretreatment on SOD activity in H_2_O_2_-stimulated SAECs. SOD activities were assessed by measuring formazan production at 450 nm as described in the Methods. Samples were obtained from supernatants of control cells, of cells stimulated with 100 μM H_2_O_2_ alone, or of cells pretreated with 1, 5 or 10 μM CAM for 72 h before stimulation with 100 μM H_2_O_2_ for 1.5 h. Data are presented as means ± SD of three to six independent experiments. ^**^p < 0.01 vs. control cells, ^##^*p* < 0.01 vs. cells stimulated with H_2_O_2_ alone, ^†^*p* < 0.05 vs. cells pretreated with 10 μM CAM

We next examined whether CAM could alter CAT and SOD protein levels in SAECs after treatment with H_2_O_2_ (100 μM) for 1.5 h. As shown in Figs. 4 and 5, both CAT and SOD activities were decreased in H_2_O_2_-treated cells. Nevertheless, pretreatment with a low concentration of CAM (1 or 5 μM) for 72 h significantly increased those activities compared with H_2_O_2_ treatment alone (*p* < 0.01). On the other hand, pretreatment with a high concentration of CAM (10 μM) for 72 h had no influence on both activities decreased by H_2_O_2_ treatment. There were also significant differences in the H_2_O_2_-induced CAT and SOD activities between the low- and high-concentration CAM groups (*p* < 0.05 or *p* < 0.01).

### Effects of CAM pretreatment on H_2_O_2_-induced HO-1 levels and HO-1 mRNA expression in SAECs

**Fig. 6:**
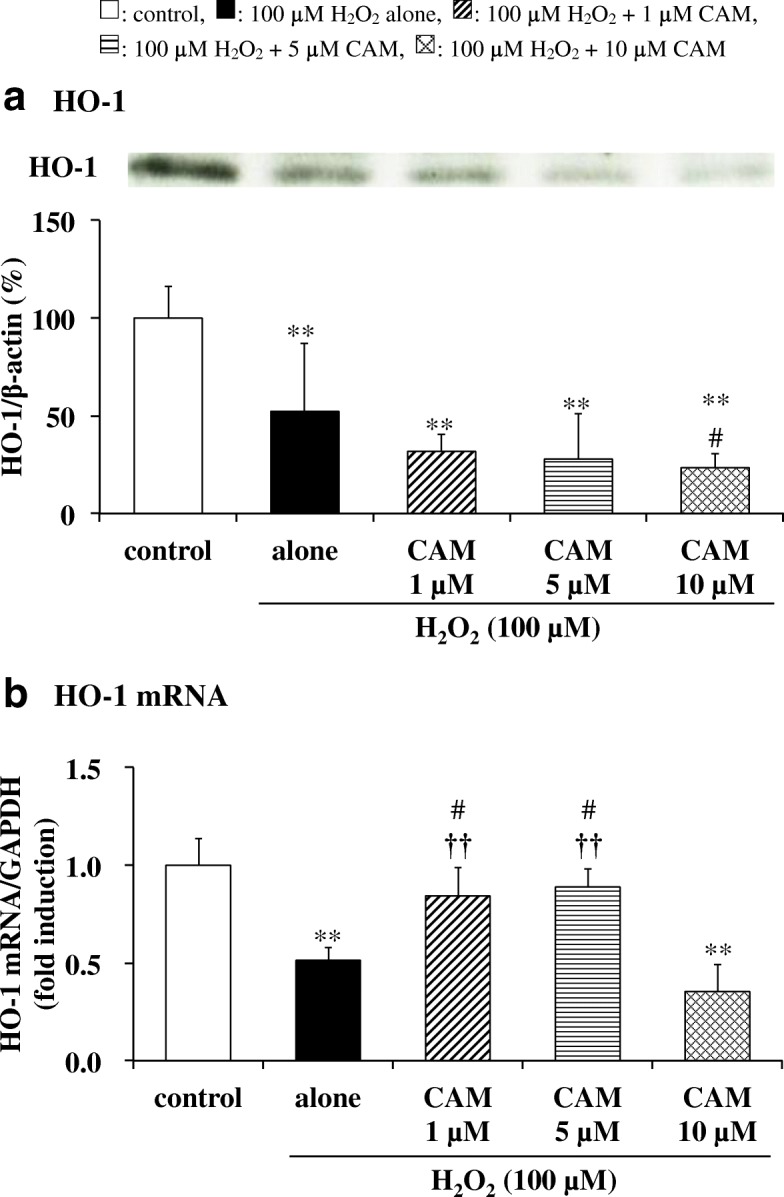
Effects of CAM pretreatment on HO-1 activation (**a**) and mRNA expression (**b**) in H_2_O_2_-stimulated SAECs. In panel **a**, HO-1 protein levels were detected by Western blotting (upper, representative blot images; lower, quantification of bands). In panel **b**, HO-1 mRNA expression was measured using real-time RT-PCR. Samples were obtained from cell pellets (**a**) or cDNA (**b**) of control cells, of cells stimulated with 100 μM H_2_O_2_ alone, or of cells pretreated with 1, 5 or 10 μM CAM for 72 h before stimulation with 100 μM H_2_O_2_ for 0.5 or 1 h, respectively. The data in panel **a** are expressed as the HO-1/β-actin ratio. Data are presented as means ± SD of three independent experiments. ^**^p < 0.01 vs. control cells, ^#^*p* < 0.05 vs. cells stimulated with H_2_O_2_ alone, ^††^*p* < 0.01 vs. cells pretreated with 10 μM CAM

Incubation with H_2_O_2_ for 0.5 h significantly decreased the HO-1 level in SAECs compared to untreated cells (*p* < 0.01). Pretreatment with CAM (1 or 5 μM) for 72 h had no effect on HO-1 levels in H_2_O_2_-treated cells (Fig. 6a, *p* > 0.05 vs. H_2_O_2_ treatment alone). When SAECs were pretreated with 10 μM CAM for 72 h, the decrease in HO-1 level induced by treatment with 100 μM H_2_O_2_ was rather exacerbated (*p* < 0.05).

HO-1 mRNA expression was also significantly suppressed by H_2_O_2_ treatment (100 μM) for 1 h. In contrast to its effects on the HO-1 level, pretreatment with 1 or 5 μM CAM, but not with 10 μM CAM, for 72 h significantly increased HO-1 mRNA expression in H_2_O_2_-treated cells compared to H_2_O_2_ treatment alone (*p* < 0.01) (Fig. 6b). As expected, there was a significant difference in HO-1 mRNA expression in H_2_O_2_-treated cells between the low- and high-concentration CAM groups (*p* < 0.01).

### Effects of CAM on H_2_O_2_-induced ERK phosphorylation in SAECs

**Fig. 7:**
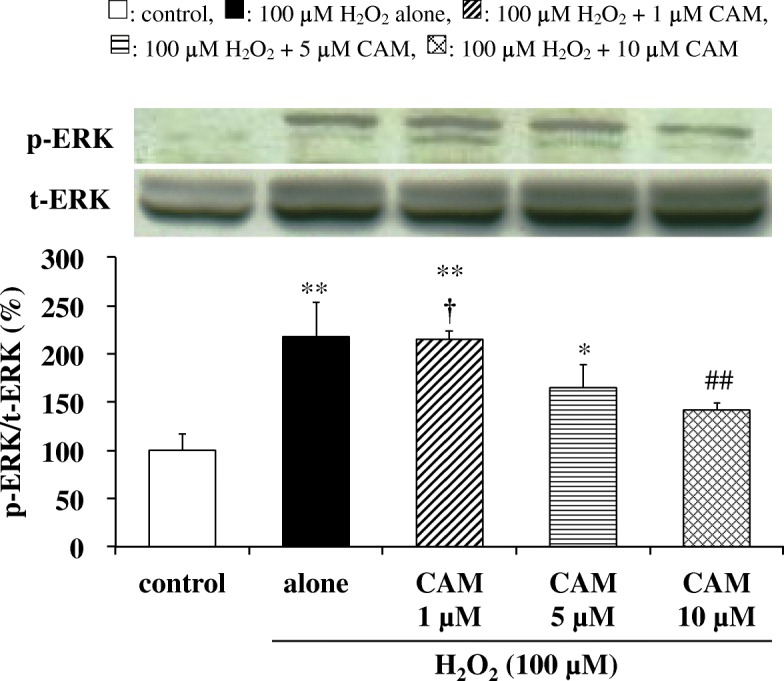
Effects of CAM pretreatment on p-ERK expression in SAECs stimulated with H_2_O_2_. p-ERK and t-ERK protein levels were detected by Western blotting (upper, representative blot images; lower, quantification of bands). Samples were obtained from cell pellets of control cells, of cells stimulated with 100 μM H_2_O_2_ alone, or of cells pretreated with 1, 5, or 10 μM CAM for 72 h before stimulation with 100 μM H_2_O_2_ for 0.5 h. The data are expressed as the p-ERK/t-ERK ratio. Data are presented as means ± SD of three independent experiments. ^*^p < 0.05, ^**^p < 0.01 vs. control cells, ^##^*p* < 0.01 vs. cells stimulated with H_2_O_2_ alone, ^†^*p* < 0.05 vs. cells pretreated with 10 μM CAM

The effects of CAM on H_2_O_2_-induced ERK phosphorylation in SAECs were investigated using Western blot analysis. Exposure of SAECs to H_2_O_2_ (100 μM) for 0.5 h significantly increased ERK phosphorylation versus control cells, and this increase in ERK phosphorylation was maintained with treatment of 1 or 5 μM CAM for 72 h before H_2_O_2_ treatment. However, pretreatment with 10 μM CAM for 72 h resulted in a significant decline in H_2_O_2_-induced ERK phosphorylation (Fig. 7, *p* < 0.01 vs. H_2_O_2_ treatment alone). As expected, pretreatment with a high concentration (10 μM) of CAM for 72 h also significantly decreased H_2_O_2_-induced ERK phosphorylation compared to pretreatment with a low concentration (1 μM) of CAM for 72 h (*p* < 0.01).

### Effects of U0126 pretreatment on the viability of H_2_O_2_-treated cells

**Fig. 8:**
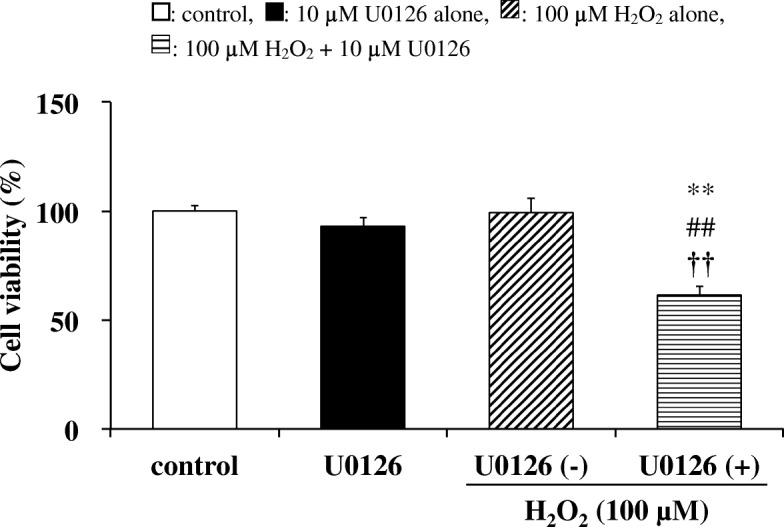
Effects of U0126 on cell viability in SAECs stimulated with H_2_O_2_. Cell viability was assessed by measuring formazan production from viable cells (at 450 nm) as described in the Methods. Samples were obtained from control cells, from cells pretreated with 10 μM U0126 alone for 0.5 h, or from cells pretreated with or without 10 μM U0126 for 0.5 h before stimulation with 100 μM H_2_O_2_ for 3 h. Data are presented as means ± SD of three independent experiments. ^**^p < 0.01 vs. control cells, ^##^*p* < 0.01 vs. cells pretreated with 10 μM U0126 alone, ^††^*p* < 0.01 vs. cells pretreated without 10 μM U0126 before H_2_O_2_ stimulation (cells stimulated with H_2_O_2_ alone)

The relationship between suppression of ERK phosphorylation and cell viability was examined using U0126, an ERK inhibitor. When SAECs were treated with U0126 (10 μM) or H_2_O_2_ (100 μM) for 0.5 or 3 h, respectively, no significant changes in cell viability were observed (Fig. 8). However, in cells pretreated with U0126 before exposure to H_2_O_2_, cell viability was significantly reduced as compared with H_2_O_2_-treatment alone. This indicates that suppression of ERK phosphorylation promotes a decrease in cell viability following H_2_O_2_ treatment.

## Discussion

It is well known that low-dose, long-term macrolide therapy is effective against chronic inflammatory airway diseases [1–5]. As shown in Fig. 1, we previously demonstrated that low-dose (1 or 5 μM), long-term (72 h) CAM treatment inhibited H_2_O_2_-induced reduction of the GSH/GSSG ratio in SAECs, via the maintenance of GSH levels through an effect on γ-GCS expression [8]. In contrast to the effect of low-dose CAM, reduction of the GSH/GSSG ratio was not prevented by long-term pretreatment of SAECs with a high (10 μM) CAM concentration. In general, H_2_O_2_-induced cytotoxicity is thought to be controlled by the ROS scavenging system, including cellular antioxidant enzymes [13], in addition to increasing γ-GCS expression [8, 22]. However, the influence of CAM on antioxidant enzyme activities remains unclear. Therefore, in the present study, we first examined the influence of CAM against antioxidant enzymes in SAECs, which are the main cell type involved in chronic airway diseases.

Our study confirmed that CAM inhibited the H_2_O_2_-induced reduction of GPx-1 activity with low-dose treatment, which suppressed the decrease in the GSH/GSSG ratio, but not with high-dose treatment (Fig. 2a). In addition, this inhibition was associated with increased expression of GPx-1 mRNA, indicating that an increase in GPx-1 activity occurs at the transcriptional level (Fig. 2b). GPx-1 promotes GSH oxidation to the GSSG form, and protects cells from H_2_O_2_-induced cytotoxicity. Furthermore, the lower activity of GR under an oxidative condition favors the accumulation of GSSG in cells. The deficiency of GR is characterized by the sensitivity of membranes to H_2_O_2_ and contributes to oxidative stress, which plays a key role in the pathogenesis of many diseases including chronic airway diseases [23]. Pretreatment with a low concentration (1 or 5 μM) of CAM also inhibited the H_2_O_2_-induced reduction of GR activity (Fig. 3); thus, it is suggested that the GSSG produced by GPx-1 was returned to GSH effectively by a recycling reaction involving GR. H_2_O_2_ reduced CAT activity in a manner that was similar to GPx-1, with CAM pretreatment ameliorating the effects of H_2_O_2_ (Fig. 4). In general, the binding affinity for H_2_O_2_ is considered to be higher in GPx-1 because the Km value of GPx-1 is smaller than that of CAT [24]. Therefore, under the conditions of the present study, it is thought that GPx-1 may exert a greater effect than CAT for H_2_O_2_ elimination in SAECs. That is, the 100 μM H_2_O_2_ concentration used for inducing cytotoxicity is considered to be low as a substrate of CAT. However, regardless of whether or not CAT is involved in the elimination reaction of H_2_O_2_, it is important that low-dose CAM suppresses the H_2_O_2_-induced decrease in CAT activity. During inflammatory conditions in the lower respiratory tract, neutrophil-derived H_2_O_2_ in the respiratory tract viscous fluid is reported to reach a concentration of 50 μM [25]. Since H_2_O_2_ is also produced directly from bronchial epithelial cells following stimulation with pollutants such as bacteria and LPS [26], it is thought that the H_2_O_2_ concentration (100 μM) used in this study reflects conditions observed during chronic inflammatory airway diseases. Therefore, it is thought that GPx-1 is mainly involved in the elimination of H_2_O_2_, even in clinical settings. As shown in Figs. 4 and 5, pretreatment with 1 or 5 μM CAM for 72 h showed inducible effects on CAT activity as well as SOD activity, respectively. These results indicate that low-dose CAM promotes an antioxidant effect against oxidative stress in SAECs.

Oxidative stress liberates heme from heme-proteins such as hemoglobin and cytochrome, and HO-1 catalyzes the degradation of heme to produce ferrous iron, carbon monoxide and biliverdin, the latter of which is subsequently converted into bilirubin [27, 28]. Carbon monoxide is involved in the regulation of anti-inflammation [29]. Biliverdin and bilirubin, which can scavenge peroxyl radicals, are potent cytoprotective antioxidants [30]. However, studies have shown that HO-1 overexpression (10 to 15 times the normal amount) increases ROS levels, thereby inducing cell death [31, 32]. In this study, HO-1 protein levels were significantly decreased by treatment with H_2_O_2_ in SAECs, and CAM failed to elevate the level (Fig. 6a). In contrast, the expression of HO-1 mRNA was significantly increased to the level of control cells by pretreatment with low concentrations (1 or 5 μM) of CAM for 72 h (Fig. 6b). The change in HO-1 mRNA expression brought about by low CAM concentrations in this study appears to be in the opposite direction to the change in HO-1 protein level. In contrast, another study showed that the addition of 100 μM H_2_O_2_ alone increased both HO-1 protein and mRNA levels in MC3T3-E1 cells [33]. The differences in HO-1 protein and mRNA levels between the present and previous studies are attributed to differences in the experimental cell type and the H_2_O_2_ treatment time. In regards to the exposure condition of oxidative stress, under our mild oxidative stress condition (H_2_O_2_ treatment for 30–60 min), anti-oxidative substances such as HO-1 can function to adjust ROS to normal levels [34]. Therefore, it is suggested that the rate at which HO-1 as an antioxidant removes H_2_O_2_ is faster than that at which low CAM concentrations increases the expression of HO-1 mRNA to normalize HO-1 protein levels. On the other hand, oxidative stress substantially increases the levels of HO-1 and HO-1 mRNA and causes cytotoxicity under a sustained oxidative stress condition of long-term H_2_O_2_ exposure (incubation for 0.5–14 days) [31–33]. In any case, it was clearly shown that low CAM concentrations induce HO-1 mRNA expression, resulting in protection from oxidative injury. This is a potent benefit that contributes to the anti-inflammatory action of low-dose CAM. In general, an HO-1 inducer such as hemin stimulates the expression of HO-1 mRNA and upregulates HO-1 [35, 36]. However, in the antioxidant pathway of dimethyl fumarate, another HO-1 inducer, there is a time lag between increases in expression of HO-1 and HO-1 mRNA, and the expression of both is regulated by the concentration and treatment time of dimethyl fumarate [37]. Thus, it is necessary to consider whether other HO-1 inducers show the same effect (including concentration-dependent differences) as low concentrations of CAM. In addition, hydroxyl radical may be generated from H_2_O_2_ by ferrous iron; however, it is thought that biliverdin or bilirubin could function in scavenging hydroxyl radical [30].

In patients with COPD, blood GSH level [38, 39] and SOD [40, 41], CAT [42, 43] and GPx-1 [41, 44] activities were significantly reduced compared to healthy subjects. Also, in patients with bronchial asthma, SOD [45] activity or GPx-1 and GR activities [46] are reported to decrease with decreasing GSH value when exposed to antigens or under symptom deteriorating conditions. Furthermore, it was shown that the GSH level and GPx-1 activity in sputum were decreased in adult patients with cystic fibrosis [47]. These findings strongly suggest that a reduction in antioxidant enzyme activity is involved in the pathogenesis mechanism of chronic inflammatory respiratory diseases [48–50]. Our present study revealed that treatment of SAECs with a low concentration of CAM increased GPx-1, GR, CAT and SOD activities. Moreover, it stimulated the mRNA expressions of GPx-1 and HO-1. It has been reported that all of these cellular antioxidant enzymes and mRNAs are induced via the Nrf2-mediated pathway [51–53]. Nrf2 is a critical transcription factor for protecting cells from oxidative injury. In our previous report, we described that long-term (72 h) treatment with low concentrations (1 and 5 μM) of CAM activates Nrf2 mRNA expression, inhibiting the H_2_O_2_-induced reduction of the GSH/GSSG ratio in SAECs through an increase in γ-GCS expression [8]. These findings suggest that low CAM concentrations reduced oxidative stress in SAECs by inducing the expression and activation of antioxidant protein via Nrf2 signaling, which enhances GSH levels by activating GSH synthesis. The α, β-unsaturated ketone structure leads to Nrf2 activation and acts as an inducer of antioxidant response element genes [54]. However, CAM does not have a known Nrf2-binding moiety in its chemical structure. To better understand the involvement of Nrf2 in antioxidant protein expression after treatment with CAM, future studies employing knock-down and inhibitors of Nrf2 are needed.

It was suggested that treatment with low concentrations (1 and 5 μM) of CAM enhances protective responses by the intracellular H_2_O_2_ elimination system. On the other hand, this effect was not observed in the treatment with high-dose (10 μM) CAM, and cell viability decreased as previously reported [8]. In order to clarify the cause of decreased cell viability by high CAM concentration, the effect of CAM on the phosphorylation of ERK, which is a MAP kinase pathway involved in cell proliferation, was investigated. Treatment of SAECs with low CAM concentrations for 72 h did not show any significant change in ERK phosphorylation. However, the phosphorylation was significantly decreased by treatment with high-dose CAM, indicating the possibility that cell proliferation was suppressed (Fig. 7). However, the relationship between inhibition of cell proliferation and decrease in cell viability has been unclear. Therefore, the effect on cell viability was examined using U0126, an ERK inhibitor [55]. When U0126 and H_2_O_2_ were used in combination, a strong decrease in cell viability was induced (Fig. 8). Therefore, it was revealed that suppression of cell proliferation is largely involved in the reduction of cell viability. The mode of cell death by long-term treatment with high-dose CAM can be considered as either apoptosis or necrosis. In SAECs, treatment with H_2_O_2_ (75 μM) has been reported to induce apoptosis [56]. However, we observed that CAM had no effects on caspase-3 activity and mitochondrial membrane potential in SAECs (see Additional files 5 and 6). Furthermore, it was previously shown that 72 h pretreatment with 10 μM CAM maintained H_2_O_2_-induced NF-κB activation in SAECs [8]. Therefore, there is a high possibility that anti-apoptotic factors are produced via NF-κB activation [57, 58], thereby enabling necrosis [59]. Also, as reported in the previous study [8], the levels of intracellular CAM gradually changed over time and did not plateau until after 72 h incubation of the cells with CAM. The results showed that cells incubated with 10 μM CAM did not reach concentrations of more than twice that of cells incubated with 5 μM CAM; however, significantly higher intracellular CAM concentrations were observed. Since EM has been found to exacerbate the oxidant/antioxidant balance in cells when its concentration in the extracellular fluid exceeds 6.8 μM [60], it is possible that the same changes occur in cells during pretreatment with 10 μM CAM, via an unknown mechanism. Necrosis induced by the suppression of ERK phosphorylation might be involved in this mechanism.

There is no evidence to indicate whether the exposure of cells to CAM for 72 h can be a model for the effect of long-term administration of CAM in the clinical setting. Furthermore, it is difficult to predict its effects in clinical settings based on its observed effects on cells. Nevertheless, low-dose, long-term clinical administration of CAM is thought to enhance anti-oxidative defense reactions during periods of oxidative stress.

## Conclusions

This study showed that pretreatment of SAECs with low-dose, long-term CAM resulted in increased activities of GPx-1, GR, SOD, CAT, HO-1 and mRNA expressions of GPx-1 and HO-1 after treatment with H_2_O_2_. Activation of transcription factor Nrf2 by low-dose CAM may be involved in the increased activity of these antioxidant enzymes. On the other hand, pretreatment with high-dose CAM suppressed the phosphorylation of ERK involved in cell proliferation, resulting in decreased cell viability. These data indicate that CAM is efficacious against oxidative stress-induced cell dysfunction under low-dose, long-term treatment conditions. Although it is not possible to directly translate pharmacological effects on cells into therapeutic effects in clinical practice, consistent with the previous report [8], the present study presents evidence as to why low-dose, long-term macrolide therapy is effective against chronic inflammatory airway diseases.

## Additional files

Additional file 1: Effects of CAM pretreatment on IL-8 protein and mRNA levels in H_2_O_2_-stimulated SAECs. (PDF 118 kb)

Additional file 2: Effects of CAM on cell viability in SAECs stimulated with H_2_O_2_. (PDF 82 kb)

Additional file 3: Effects of CAM on cell viability in SAECs. (PDF 61 kb)

Additional file 4: Effects of H_2_O_2_ on cell viability in SAECs. (PDF 70 kb)

Additional file 5: Caspase-3 activation induced by H_2_O_2_ after CAM pretreatment in SAECs. (PDF 74 kb)

Additional file 6: Mitochondrial membrane potential induced by H_2_O_2_ after CAM pretreatment in SAECs. (PDF 73 kb)

## Abbreviations

ANOVA: One-way analysis of variance
CAM: Clarithromycin
CAT: Catalase
COPD: Chronic obstructive pulmonary disease
DMSO: Dimethyl sulfoxide
DPBS: Dulbecco’s phosphate-buffered saline
DTNB: 5,5′-Dithiobis(2-nitrobenzoic acid)
EDTA: Ethylenediaminetetraacetic acid
EM: Erythromycin
ERK: Extracellular signal regulatory kinase
GAPDH: Glyceraldehyde-3-phosphate dehydrogenase
GPx: Glutathione peroxidase
GR: Glutathione reductase
GSH: Glutathione
GSSG: Oxidized glutathione
H_2_O_2_: Hydrogen peroxide
HO: Heme oxygenase
IL: Interleukin
JNK: c-jun N-terminal kinase
Km: Michaelis constant
LPS: Lipopolysaccharide
MAPK: Mitogen activated protein kinase
NADPH: Reduced nicotinamide adenine dinucleotide phosphate
NBT: Nitrobluetetrazolium
Nrf2: Nuclear factor erythroid 2-related factor 2
PBS: Phosphate-buffered saline
PCR: Polymerase chain reaction
p-ERK: Phosphorylated ERK
ROS: Reactive oxygen species
SABM: Small airway basal medium
SAEC: Human small airway epithelial cell
SAGM: Small airway growth medium
SOD: Superoxide dismutase
WST-8: 2-(2-Methoxy-4-nitrophenyl)-3-(4-nitrophenyl)-5-(2,4-disulfophenyl)-2H-tetrazolium
γ-GCS: γ-Glutamylcysteine synthetase
