# Supplementary material for: Low concentrations of clarithromycin upregulate cellular antioxidant enzymes and phosphorylation of extracellular signal-regulated kinase in human small airway epithelial cells
Source: J Pharm Health Care Sci. 2018 Sep 3;4:23. doi: 10.1186/s40780-018-0120-4 (PMC6120091; doi:10.1186/s40780-018-0120-4)
Supplement: Supplementary file 1 — Effects of CAM pretreatment on IL-8 protein and mRNA levels in H2O2-stimulated SAECs. (PDF 118 kb) [file 40780_2018_120_MOESM1_ESM.pdf]

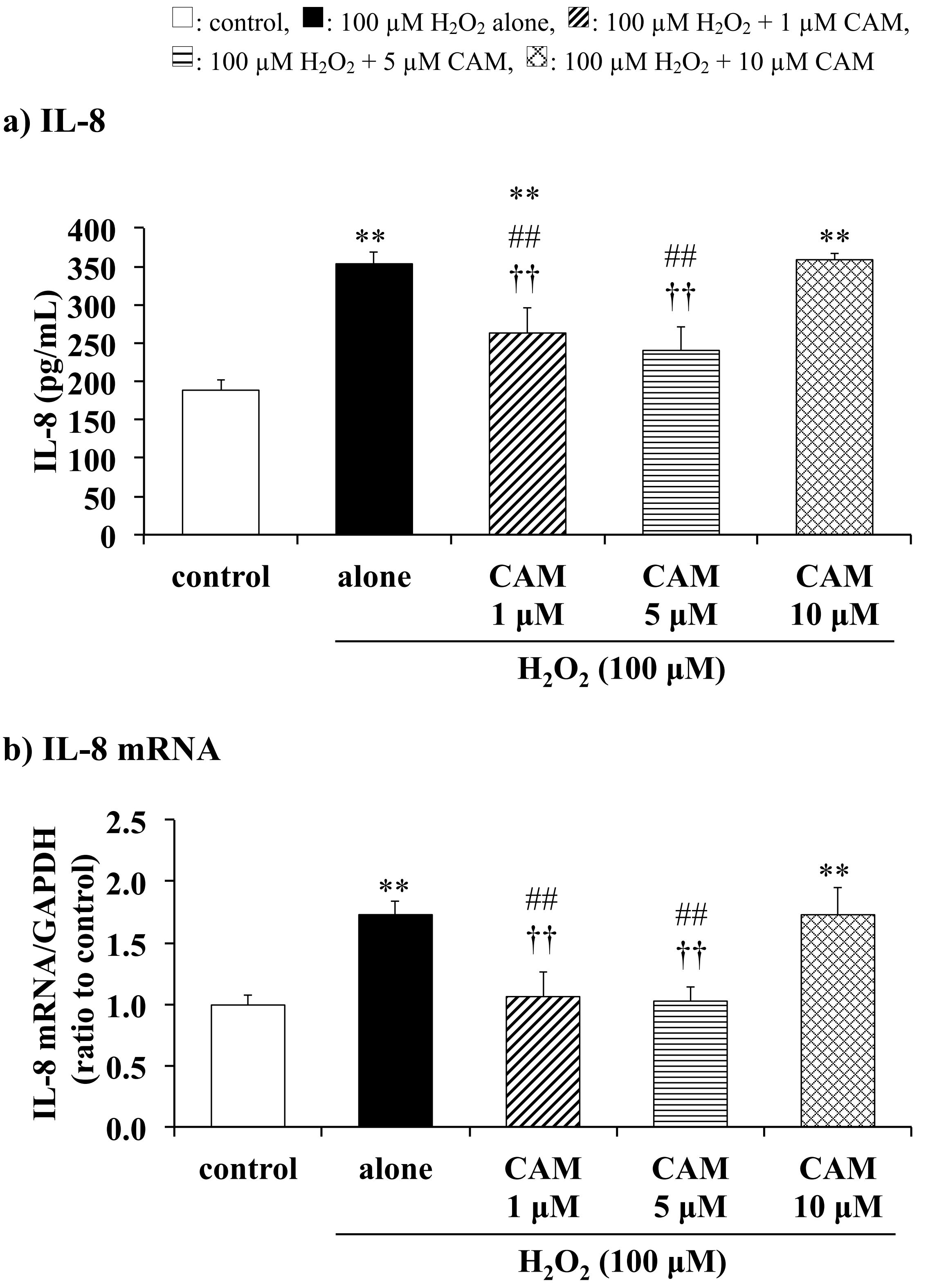

Additional file 1 Effects of CAM pretreatment on IL-8 protein (**a**) and mRNA (**b**) levels in H_2O_2-stimulated SAECs.

In panel **a**, levels of IL-8 protein were measured using the ELISA method. In panel **b**, IL-8 mRNA expression was measured using real-time RT-PCR. Samples were obtained from supernatants (**a**) or cDNA (**b**) of control cells, of cells stimulated with 100 μM H_2O_2 alone, or of cells pretreated with 1 μM, 5 μM or 10μM CAM 72 h before stimulation with 100 μM H_2O_2 for 4 or 1 h, respectively. Data are presented as means ± SD of three independent experiments. $^{*}p<0.05$, $^{**}p<0.01$ vs. control cells, $^{\#\#}p<0.01$ vs. cells stimulated with H_2O_2 alone, $^{\dagger\dagger}p<0.01$ vs. cells pretreated with 10 μM CAM
