# Supplementary material for: Low concentrations of clarithromycin upregulate cellular antioxidant enzymes and phosphorylation of extracellular signal-regulated kinase in human small airway epithelial cells
Source: J Pharm Health Care Sci. 2018 Sep 3;4:23. doi: 10.1186/s40780-018-0120-4 (PMC6120091; doi:10.1186/s40780-018-0120-4)
Supplement: Supplementary file 2 — Effects of CAM on cell viability in SAECs stimulated with H2O2. (PDF 82 kb) [file 40780_2018_120_MOESM2_ESM.pdf]

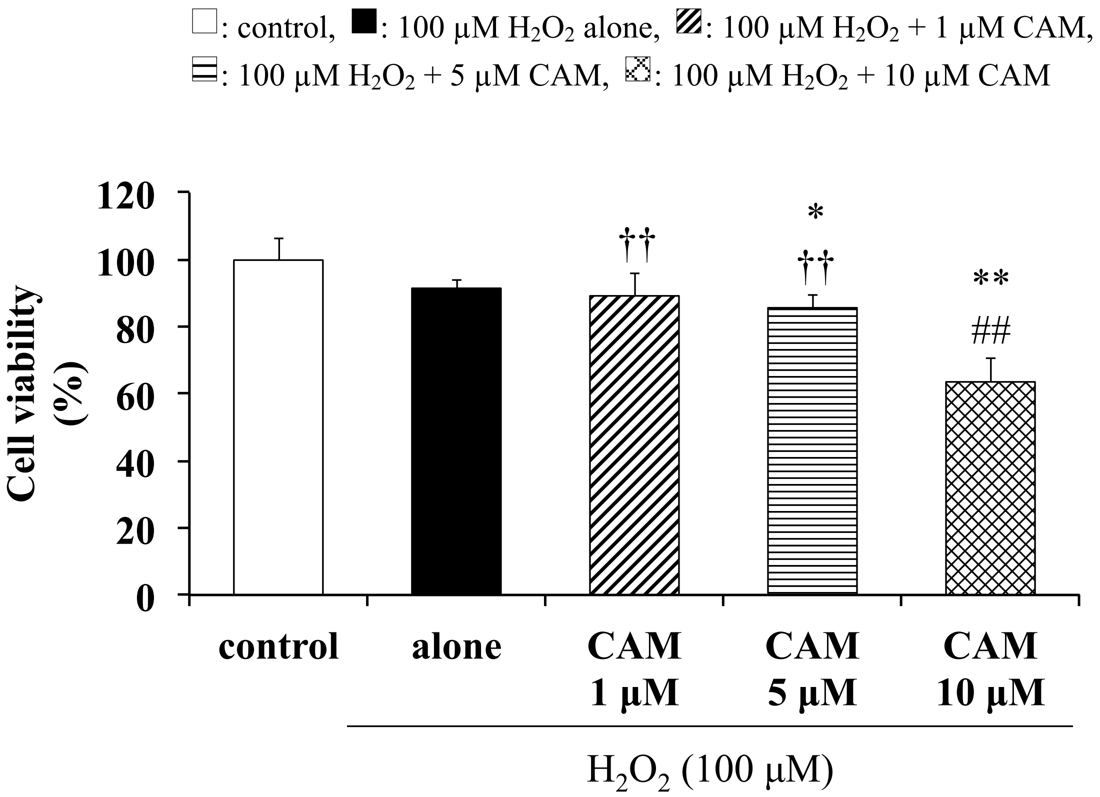

Additional file 2 Effects of CAM on cell viability in SAECs stimulated with H_2O_2. Samples were obtained from control cells, from cells stimulated with 100 µM H_2O_2 alone, or from cells pretreated with 1 µM, 5 µM, and 10 µM CAM for 72 h before stimulation with 100 µM H_2O_2 for 3 h. Data are presented as means ± SD of six independent experiments. *$p<0.05$, **$p<0.01$ vs. control cells, ##$p<0.01$ vs. cells stimulated with H_2O_2 alone, ††$p<0.01$ vs. cells pretreated with 10 µM CAM.
