# Supplementary material for: Low concentrations of clarithromycin upregulate cellular antioxidant enzymes and phosphorylation of extracellular signal-regulated kinase in human small airway epithelial cells
Source: J Pharm Health Care Sci. 2018 Sep 3;4:23. doi: 10.1186/s40780-018-0120-4 (PMC6120091; doi:10.1186/s40780-018-0120-4)
Supplement: Supplementary file 3 — Effects of CAM on cell viability in SAECs. (PDF 61 kb) [file 40780_2018_120_MOESM3_ESM.pdf]

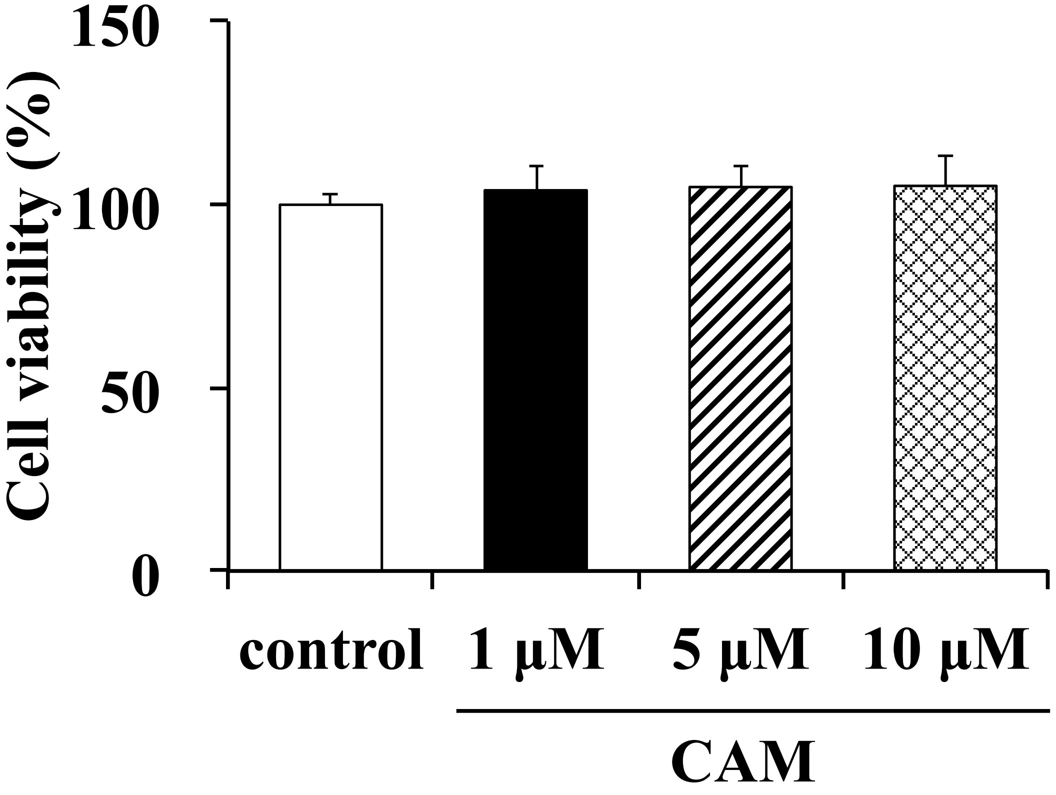

Additional file 3 Effects of CAM on cell viability in SAECs.

Cells were incubated with 1 µM, 5 µM or 10 µM CAM for 72 h. Cell viability was assessed by measuring formazan production from viable cells (at 450 nm) as described in the Materials and Methods. Data are presented as means ± SD of three independent experiments.
