# Supplementary material for: Low concentrations of clarithromycin upregulate cellular antioxidant enzymes and phosphorylation of extracellular signal-regulated kinase in human small airway epithelial cells
Source: J Pharm Health Care Sci. 2018 Sep 3;4:23. doi: 10.1186/s40780-018-0120-4 (PMC6120091; doi:10.1186/s40780-018-0120-4)
Supplement: Supplementary file 4 — Effects of H2O2 on cell viability in SAECs. (PDF 70 kb) [file 40780_2018_120_MOESM4_ESM.pdf]

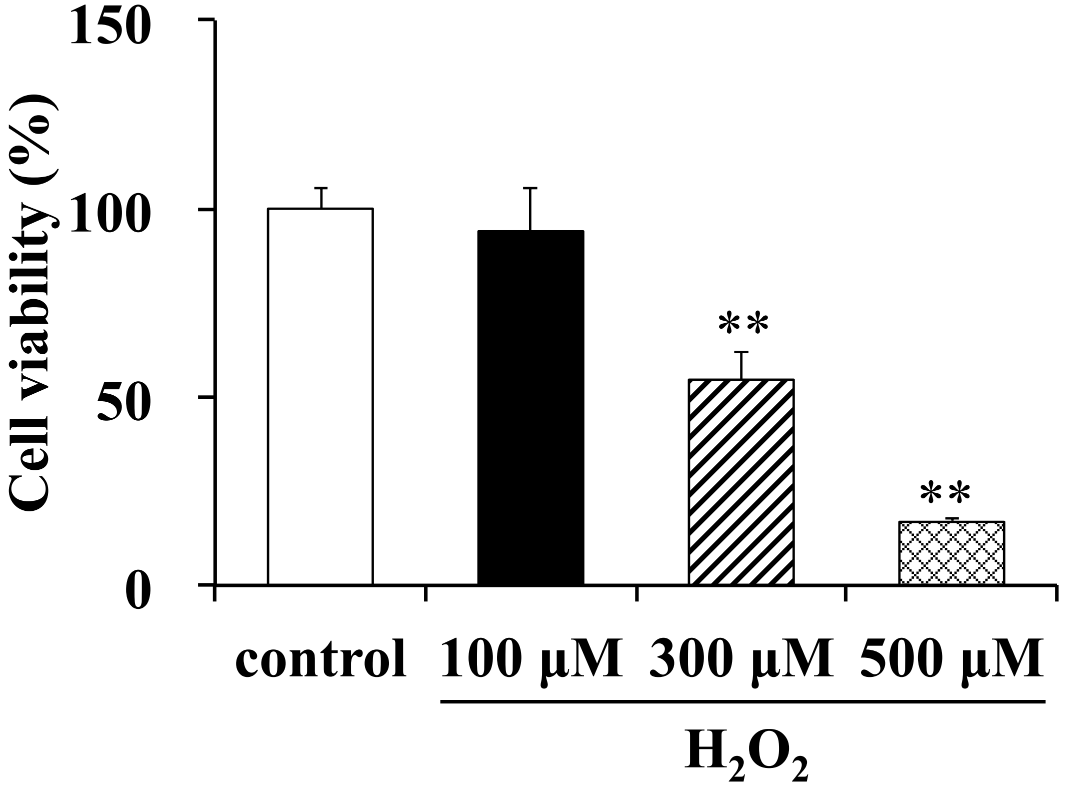

Additional file 4 Effects of H_2O_2 on cell viability in SAECs.

Cells were incubated with 100 µM, 300 µM or 500 µM H_2O_2 for 6 h. Cell viability was assessed by measuring formazan production from viable cells (at 450 nm) as described in the Materials and Methods. Data are presented as means ± SD of three independent experiments. **$p < 0.01$ vs. control cells
