# Supplementary material for: Low concentrations of clarithromycin upregulate cellular antioxidant enzymes and phosphorylation of extracellular signal-regulated kinase in human small airway epithelial cells
Source: J Pharm Health Care Sci. 2018 Sep 3;4:23. doi: 10.1186/s40780-018-0120-4 (PMC6120091; doi:10.1186/s40780-018-0120-4)
Supplement: Supplementary file 6 — Mitochondrial membrane potential induced by H2O2 after CAM pretreatment in SAECs. (PDF 73 kb) [file 40780_2018_120_MOESM6_ESM.pdf]

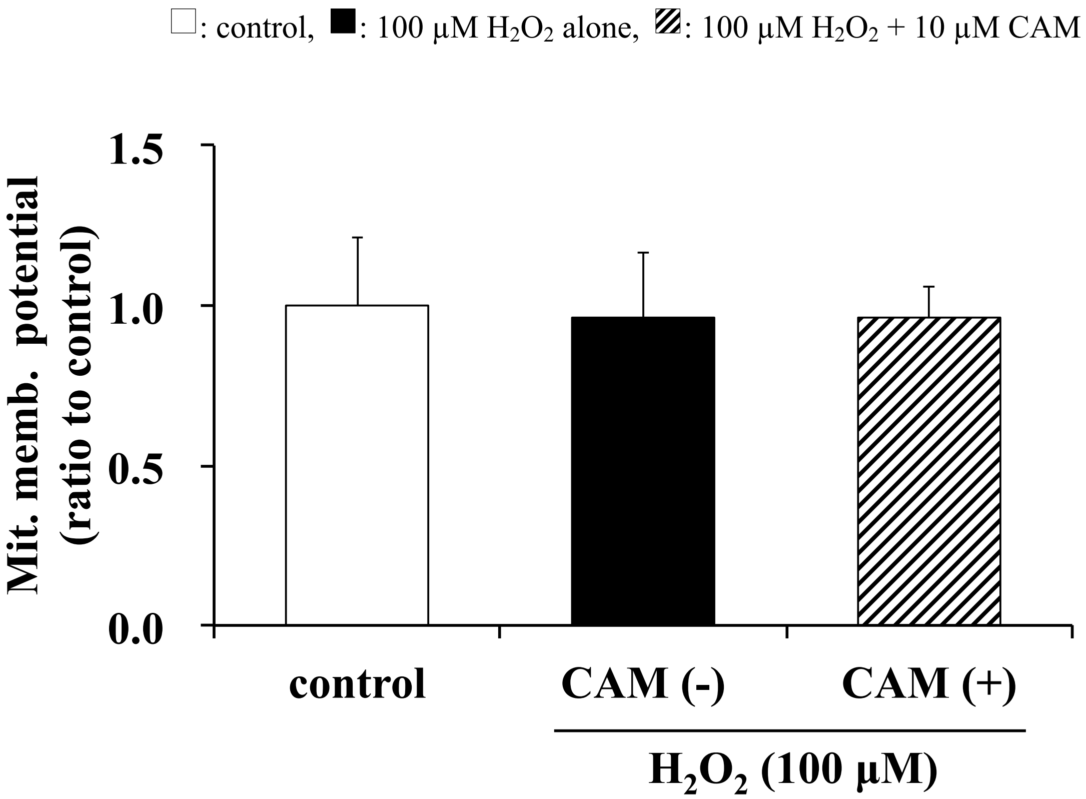

Additional file 6 Mitochondrial membrane potential induced by H_2O_2 after CAM pretreatment in SAECs.

Mitochondrial membrane potential was analyzed by using a MitoLight apoptosis detection kit and by flow cytometry. Samples were obtained from control cells, or from cells pretreated with or without 10 µM CAM for 72 h before stimulation with 100 µM H_2O_2 for 3 h. Relative fluorescence of control cells was set to 1.0. Data are presented as means ± SD of three independent experiments.
